# Reduced Steroid Metabolites Identify Infection-Prone Children in Two Independent Pre-Birth Cohorts

**DOI:** 10.3390/metabo12111108

**Published:** 2022-11-13

**Authors:** Nicole Prince, Min Kim, Rachel S. Kelly, Joann Diray-Arce, Klaus Bønnelykke, Bo L. Chawes, Mengna Huang, Ofer Levy, Augusto A. Litonjua, Jakob Stokholm, Craig E. Wheelock, Hans Bisgaard, Scott T. Weiss, Jessica A. Lasky-Su

**Affiliations:** 1 Channing Division of Network Medicine, Department of Medicine, Brigham and Women’s Hospital and Harvard Medical School, Boston, MA 02115, USA; 2 Copenhagen Prospective Studies on Asthma in Childhood (COPSAC), Herlev and Gentofte Hospital, University of Copenhagen, 2820 Gentofte, Denmark; 3 Precision Vaccines Program, Division of Infectious Diseases, Boston Children’s Hospital, Boston, MA 02115, USA; 4 Division of Pediatric Pulmonary Medicine, Department of Pediatrics, Golisano Children’s Hospital and University of Rochester Medical Center, Rochester, NY 14642, USA; 5 Department of Medical Biochemistry and Biophysics, Division of Physiological Chemistry 2, Karolinska Institute, 17177 Stockholm, Sweden

**Keywords:** infection proneness, respiratory infections, metabolomics, steroids, immunity

## Abstract

Recurrent respiratory infections are a leading cause of morbidity and mortality in early life, but there is no broadly accepted means to identify infection-prone children during this highly vulnerable period. In this study, we investigated associations between steroid metabolites and incident respiratory infections in two pre-birth cohorts to identify novel metabolomic signatures of early infection proneness. Children from the Vitamin D Antenatal Asthma Reduction Trial and the Copenhagen Prospective Studies on Asthma in Childhood were included, and profiling was performed on plasma samples collected at ages 1 and 6 years. Both cohorts recorded incidence of lower respiratory infections, upper respiratory infections, ear infections, and colds. Poisson regression analysis assessed the associations between 18 steroid metabolites and the total number of respiratory infections that occurred in offspring during follow-up. We found that steroid metabolites across androgenic, corticosteroid, pregnenolone, and progestin classes were reduced in children that suffered more infections, and these patterns persisted at age 6 years, generally reflecting consistency in direction of effect and significance. Our analysis suggested steroid metabolite measurement may be useful in screening for infection proneness during this critical developmental period. Future studies should clinically evaluate their potential utility as a clinical screening tool.

## 1. Introduction

Respiratory infections comprise a large proportion of the disease burden in early life [1]. While advances in modern medicine have reduced their associated morbidity and mortality, lower respiratory tract infections remain a leading cause of mortality in young children, causing nearly 4 million deaths annually and representing the number one cause of death in children under the age of 5 years [2]. Recurrence of these infections during childhood is associated with development of chronic diseases later in life [3,4] that have a significant influence on long-term health [5,6,7]. Previous research has demonstrated that some children are more susceptible to infections during this period [8], but the factors leading to increased infection proneness are not well understood. Steroids play important roles in regulating immune response [9] and represent one pathway of interest to understand this phenomenon [10,11]. The associations between steroids and early life infection susceptibility have not been investigated thoroughly. Given their immunologic relevance, steroid metabolomic profiles could hold important information to identify highly susceptible children.

Steroids are lipophilic messengers that impact a range of physiological functions relevant to lung and immune outcomes [12,13]. The role of steroids begins during the in utero period, as they are critical factors in fetal lung and immune system development [14]. Steroids continue to influence immune competency throughout life by regulating immune cell development and activity [15,16]. Despite these well-established functions, the relationships between steroid metabolites and infection proneness have yet to be systematically assessed. Metabolomic profiling in plasma can estimate the net result of many biological functions and provides an indication of physiological states directly related to phenotype [17]. Incorporating metabolomic profiling over multiple time points enhances understanding of phenotypes, and it is essential for steroids in particular, as these concentrations fluctuate throughout infancy and child development [18]. Steroid metabolites in the first year of life can reveal information relevant to the critical period of rapid postnatal immune system adaptation [19]. These early disruptions can produce long-term effects [20], and follow-up inquiry in childhood is essential to understand how these relationships may vary over time.

Using data from the Vitamin D Antenatal Asthma Reduction Trial (VDAART) and the Copenhagen Prospective Studies on Asthma in Childhood (COPSAC), we investigated associations between steroid metabolites and childhood infection proneness, utilizing the cumulative number of respiratory infections that occurred during follow-up as a proxy for infection susceptibility. We employed a discovery and replication approach to understand the connection between steroid metabolite profiles and infection proneness across two time periods: (1) during infancy (1 year, 18 months) and (2) early school age (6 years), and further investigated these relationships with respect to sex and medication use. We hypothesized that steroid metabolite profiles would differ in children that experienced a higher number of infections during early life. To our knowledge, this is the first study to investigate steroid metabolite profiles related to this outcome, and the results may hold importance as an initial step toward screening and early intervention approaches.

## 2. Materials and Methods

### 2.1. Study Populations

VDAART was a randomized, double-blind, parallel-design trial conducted at three study sites across the United States (ClinicalTrials.gov identifier: NCT00920621), and details of the study design and sample size calculation can be found in Litonjua et al. [21]. VDAART recruited pregnant mothers and randomized them to vitamin D supplementation at 4000 IU/day or placebo; all women received 400 IU/day vitamin D supplementation as part of usual pregnancy care. Blood draws were collected in EDTA tubes from children at 1 and 6 years of age, and plasma was separated through centrifugation at 2000 RPM at 4 °C and stored at −80 °C until metabolomic profiling.

COPSAC was a single-center, population-based birth cohort study, and details of the study design and sample size calculation can be found in Bisgaard, et al. [22]. Mothers were randomized to vitamin D_3_ supplementation at 2400 IU/day vs. placebo, and all women received 400 IU/day of vitamin D_3_ as part of usual pregnancy care. Women were also randomly assigned in a 1:1 ratio to receive 2.4 g per day of n–3 long-chain polyunsaturated fatty acids (LCPUFAs) in triacylglycerol form from fish oil capsules containing 55% eicosapentaenoic acid (20:5n–3, EPA) and 37% docosahexaenoic acid (22:6n–3, DHA; Incromega TG33/22, Croda, Health Care, Snaith, UK) or placebo in the form of lookalike olive oil capsules containing 72% n–9 oleic acid and 12% n–6 linoleic acid (Pharma-Tech A/S, Randers, Denmark). Blood samples were collected from children during visits at age 18 months (Heparin-Li tubes) and age 6 years (EDTA tubes); plasma was collected and stored at −80 °C until processing [23].

Metabolomic profiles were generated using Ultrahigh Performance Liquid Chromatography Tandem Mass Spectrometry (UPLC-MS/MS) by Metabolon, Inc. (Durham, NC, USA) for both the VDAART and COPSAC samples. Pooled samples and technical replicates were run to evaluate biological variability and technical variability. Details of the UPLC-MS/MS and data quality control are provided in the Supplementary Methods.

### 2.2. Clinical Outcomes

The infection proneness phenotype was based on the total number of parent-reported lower respiratory infections (LRIs), upper respiratory infections (URIs), ear infections, and colds that occurred during follow-up in children. In VDAART, data were collected through standardized questionnaires completed quarterly by parents beginning at birth until the child reached age 6 years; in COPSAC, data were recorded until age 3 years (Figure S1). Asthma status was also collected in VDAART and COPSAC during the 6-year follow-up period, and children were recorded as asthmatic if they received a formal diagnosis of asthma by a physician at any time during the first 6 years of life.

### 2.3. Analysis of Steroid Metabolite Profiles and Infection Proneness

Analyses of the associations between steroid metabolites and respiratory infection proneness were conducted with a discovery and replication approach, in which VDAART served as our discovery population and COPSAC was the replication population. Poisson regression models were used to assess associations between steroid metabolites and childhood respiratory infection proneness at each childhood time point (VDAART: age 1 year and age 6 years; COPSAC: age 18 months and age 6 years). Potential confounding factors were selected based on scientific literature and previous research considering their causal relationships with the steroid metabolites and their associations with risk of recurrent infections during childhood [24]. Models were adjusted for sex (female, male), race (White, Black, Other), ethnicity (Hispanic or Latino, Not Hispanic or Latino), study site (Boston, St. Louis, San Diego), body mass index (BMI), vitamin D level in blood at time of blood draw (nanograms of 25 hydroxyvitamin D [25(OH)D] per milliliter of blood [ng/mL]), and inhaled or oral corticosteroid (ICS, OCS) medication use within 3 months of blood draw. COPSAC was homogenous with respect to race and ethnicity, so adjustments for race, ethnicity, and study site were not included; however, for COPSAC individuals, an additional adjustment for fish oil intervention was included.

Additional models assessed the interaction between biological sex at birth and steroid metabolites. We conducted further analysis restricted to non-users. ICS/OCS use was classified as ever use or never use, and ever use was defined as any reported use of these medications during follow-up. All analyses were conducted using the glm package in R version 4.0.3 [25]. Correction for false discovery rate (FDR) was performed using the Benjamini–Hochberg Procedure. Statistical significance in the regression models was determined by setting a threshold of *p* < 0.05 after FDR correction for multiple testing. We inferred associations based on the incidence rate ratios (IRRs) produced through Poisson regression analysis.

### 2.4. Assessment of Asthma and Respiratory Infection Proneness Comorbidities

We investigated the overlap in asthma and respiratory infection proneness phenotypes in this study using logistic regression models to assess associations between asthma diagnosis by age 6 years and quartiles of respiratory infections. Individuals in each cohort were divided into quartiles based on the cumulative number of respiratory infections during follow-up. Logistic regression models in VDAART were adjusted for the same confounders as in the previously described models, excluding steroid medication use, since this is inextricably tied to asthma users. Analyses were conducted using the glm package in R version 4.0.3 [25].

## 3. Results

### 3.1. VDAART and COPSAC Populations

**Figure 1:**
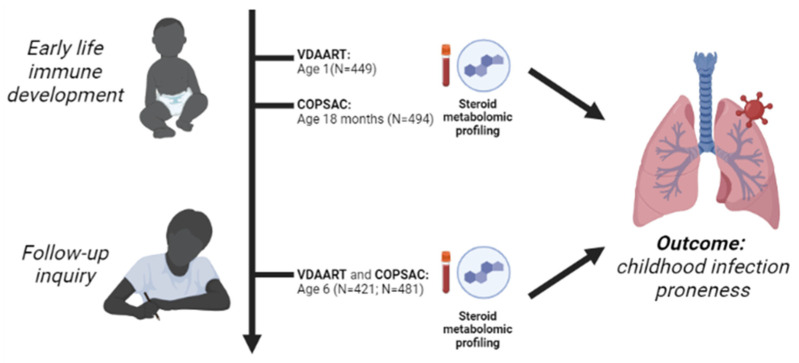
Overview of study design and analysis. A discovery and replication approach was utilized in which VDAART study participants served as the discovery cohort and COPSAC subjects served as the replication cohort. Plasma metabolomics were measured in infancy (age 1 year, age 18 months) with a further inquiry and follow-up at age 6 years to understand the associations between plasma steroid metabolites and childhood infection proneness. Image created using BioRender.

We employed a discovery and replication approach using two birth cohorts, VDAART and COPSAC, that conducted follow-up in children over the first 6 years of life (Figure 1). Table 1 summarizes the VDAART and COPSAC cohort characteristics during the two time periods of interest: (1) infancy (age 1 year in VDAART; age 18 months in COPSAC) and (2) early school age (age 6 years for both studies). Based on the suitability of blood samples for metabolomic profiling, a slightly different subset of children was available at the two time points. Two hundred and fifty-six children overlapped between the age 1 and age 6 time points in VDAART and 358 children overlapped between the time points at age 18 months and age 6 years in COPSAC. For children in VDAART, the average number of respiratory infections the occurred between birth and age 6 years was 28.3; for individuals in COPSAC, the average number of cumulative respiratory infections the occurred between birth and age 3 years was approximately 15.2.

### 3.2. Reduced Steroid Metabolite Levels Were Associated with Higher Infection Proneness

**Figure 2:**
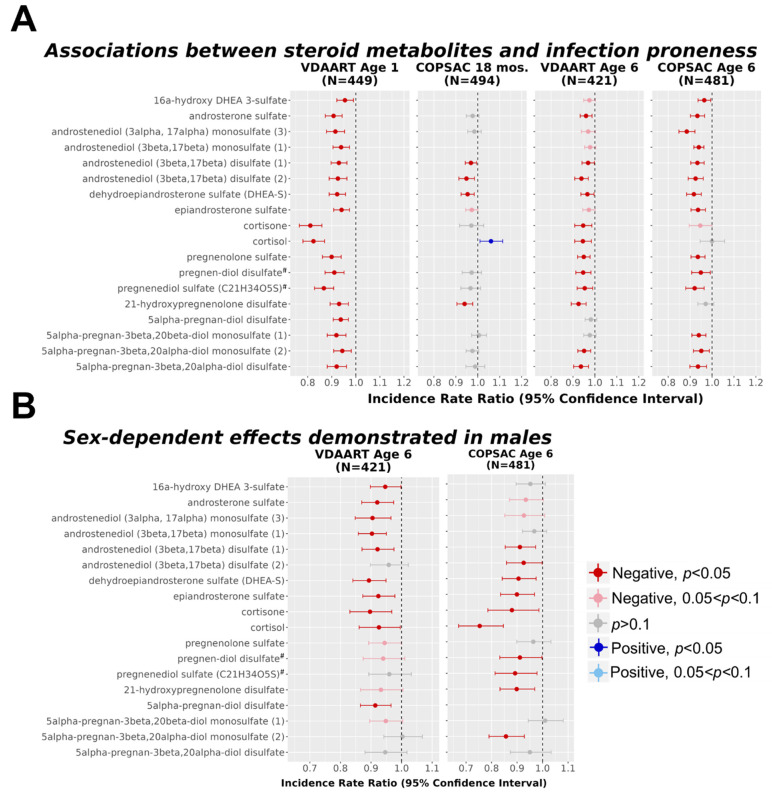
Reduced plasma steroid metabolites were associated with higher infection proneness and males demonstrated further reduction at age 6 years. (**A**) Poisson regression analysis was used to determine association between steroid metabolites and infection proneness across two time points: infancy (aged 1 year/aged 18 months) or early school age (aged 6 years). Models were adjusted for sex, race, ethnicity, study site, BMI, steroid medication use within 3 months of blood draw, and year 6 vitamin D level (italicized = covariate only in VDAART); fish oil intervention status was included as a covariate for COPSAC only; (**B**) Interaction coefficients are shown for interactions between steroid metabolites at age 6 years and sex. Metabolite names marked with a superscript hash sign (#) denote metabolite identification at tier 2 level (matching Metabolon database, details in Methods).

A total of 18 steroid metabolites were identified and met quality control standards (Table S1). The results demonstrated that reduced levels of steroids in every sub-pathway were observed in children that experienced a higher number of infections at the infancy time point in VDAART (range of *p*-values = 0.0127 to 2.33 × 10^−12^; Figure 2, Table S2). These associations were robust to a threshold of FDR < 0.05 for VDAART children at age 1 year. Fourteen steroid metabolites measured in VDAART were also measured in the corresponding COPSAC time point at age 18 months; four steroid metabolites replicated the associations observed in VDAART at a nominal significance threshold of *p* < 0.05 (range of *p*-values = 0.022 to 1.55 × 10^−3^), and three of four retained significance at an FDR threshold of 0.05. In COPSAC at age 18 months, cortisol was positively associated with infection proneness but was not robust to an FDR < 0.05 threshold.

These findings were consistent in a follow-up inquiry at age 6 for both the VDAART and COPSAC cohorts, demonstrating that reduced steroid metabolite levels remained significantly associated with high infection proneness at the later time point. Nominally significant associations were observed in 12 of the 18 steroid metabolites in VDAART (range of *p*-values = 0.035 to 4.05 × 10^−5^), and 11 of 12 associations retained significance at an FDR < 0.05 cutoff (Table S2). In VDAART, significant associations spanned all classes of steroid metabolites measured. In COPSAC, 14 of the 17 steroid metabolites measured were inversely associated with infection proneness, representing all steroid sub-pathways except corticosteroids (range of *p*-values = 0.018 to 4.28 × 10^−9^; Figure 2, Table S2); all 14 of these associations were robust to an FDR < 0.05 threshold.

Additional models were analyzed to assess the interaction of steroid metabolites with child sex, and the results demonstrated that steroid levels were further reduced in infection-prone males compared to infection-prone females (Figure 2B; Table S3). In each cohort, 10 steroid metabolites demonstrated significant reductions in males; there were 5 steroids that were consistent between the VDAART and COPSAC cohorts, including 3 androgenic steroid metabolites and 2 corticosteroid metabolites.

### 3.3. Reduced Steroid Metabolite Relationships Remained in ICS and OCS Non-Users

**Figure 3:**
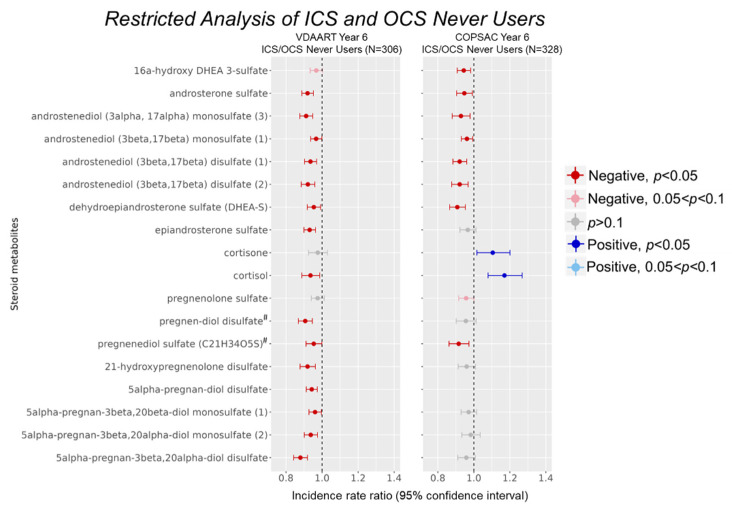
Reduced steroid levels were observed in restricted analysis of ICS and OCS non-users. ICS/OCS use between birth to age 6 was converted to a binary variable according to ever use (TRUE) or never use (FALSE). Steroid metabolite associations with infection proneness were assessed within never users of these medications to eliminate potential confounding by their use. Models were adjusted for the same covariates as in the original models. Metabolite names marked with a superscript hash sign (#) denote metabolite identification at tier 2 level (matching Metabolon database, details in Methods).

To eliminate potential confounding by ICS and OCS medication use within our cohorts, we performed analysis restricted to never users of these medications. Approximately 72.7% of VDAART and 68.2% of COPSAC age-6 individuals fell into the never use category (Table 1). Results from the restricted analysis demonstrated that 15 of 18 metabolites in VDAART retained the inverse associations observed in the overall analysis at a nominal significance threshold of *p* < 0.05 (Table S4, Figure 3). One additional androgen metabolite was borderline significant at *p* < 0.1. Significantly associated metabolites in the restricted analysis spanned all sub-classes of steroid metabolites. In COPSAC, 8 of 17 steroid metabolites produced significant negative associations at *p* < 0.05, including 7 androgenic and 1 pregnenolone steroid (Table S4, Figure 3). One pregnenolone metabolite was borderline significant at *p* < 0.1. Of the non-significant associations, six steroid metabolites were consistent in direction, although these did not reach nominal significance. In COPSAC children, cortisone and cortisol were positively associated with infection proneness (*p* = 0.035 and 8.04 × 10^−4^, respectively).

### 3.4. Infection-Prone Children Were More Likely to Be Diagnosed with Asthma

Logistic regression models evaluated associations between infection proneness quartiles and the outcome of child asthma status, and children in higher infection proneness quartiles demonstrated significantly higher risk of asthma (Table 2). In VDAART, children in the highest quartile of infection proneness had a 32% increased risk of asthma diagnosis relative to those in the lowest quartile (OR [95%CI]: 1.32 [1.31, 1.33]; *p*-value 3.71 × 10^−6^). This relationship was replicated in COPSAC and children showed a 49% increased risk of asthma (OR [95%CI]: 1.49 [1.49, 1.50]; *p*-value 1.44 × 10^−13^). There was also a significant positive association between asthma diagnosis by age 6 years and membership in the third quartile in COPSAC (OR 1.19 [1.18, 1.19], *p*-value 1.65 × 10^−3^).

Logistic regression models assessed associations between the same 18 steroid metabolites and asthma diagnosis (Table S5). A nominal threshold of *p* < 0.1 for associations between steroid metabolites and asthma was used to identify significant associations. A total of 11 steroid metabolites were consistent in direction of effect and significance for both outcomes at *p* < 0.1, and reduced levels of these 11 steroids were associated with higher numbers of infections and higher risk of asthma. Steroids commonly associated with both asthma and infection proneness spanned only androgenic and pregnenolone classes.

## 4. Discussion

In this analysis of the associations between steroid metabolites and childhood infection proneness, we found strong evidence that reduced plasma levels of endogenous steroids are present in children that experienced a higher number of infections. These relationships were consistent between infancy and follow-up inquiry at age 6 years and spanned all classes of steroid metabolites measured in metabolomic profiling. The first year of life is a critical period for immune system development [11], and alterations to steroid metabolic pathways may reflect a disrupted state in infection-prone children. While there is a component of genetic susceptibility to infection [26], much less is known regarding the specific pathways and metabolomic alterations that are distinct in infection-prone children. As steroids are intimately tied to immune response to infection [9,27,28] and are instrumental in the development of the lung and immune systems [12,13], we chose to investigate this pathway and its relationship with infection proneness. A deeper understanding of steroid metabolomic signatures could enlighten the physiological changes that are unique to this highly susceptible group and offer new screening alternatives to identify infection-prone children.

Improper infection resolution is an important public health challenge [2,9,29,30,31], but there are no broadly accepted means to identify children that exhibit higher susceptibility to infections. This study is the first to demonstrate associations across entire steroid metabolite profiles and infection proneness in multiple cohorts, and our results implicated multiple classes of steroid metabolites as novel indicators of infection proneness in children. Our findings are consistent with recent literature suggesting that early disruptions to immunity led to higher numbers of childhood infections [5,10], and these results showed that changes in steroid metabolites were distinct in children that experienced more infections. These changes could be identified at a very early age, as results were generally consistent across the infancy and age 6 time points. These results challenged the assumption that all steroids are immunosuppressive, and instead showed that reduced steroids had negative impacts, both overall and in a restricted analysis of ICS and OCS never users. Previous studies have noted differences in the role of synthetic steroids [32] and our results suggested a difference between exogenous administration and endogenous physiological levels of steroids. Furthermore, we investigated the potential effects of child sex in order to assess relevant patterns in infection-prone children, as steroids may differ by sex [33]. Males showed nominally significant reductions in steroid metabolites compared to females in both cohorts, and five steroids were consistent between VDAART and COPSAC, which may suggest males demonstrate higher infection proneness than females in early life. We also considered the influence of ICS and OCS medications as these medications may also impact infection proneness [34].

Our results further suggested the potential for a shared underlying biology between infection proneness and the development of asthma, as there is an established connection between these phenotypes in the literature [35,36,37]. Infection-prone children were more likely to be diagnosed with asthma by age six in both cohorts, and reduced steroid metabolites were associated with higher risk of both phenotypes. Eleven steroid metabolites were consistently associated with both outcomes at *p* < 0.1, which may provide further evidence for a role of pregnenolones and androgenic steroids in the development of these phenotypes. Notably, endogenous corticosteroid metabolite levels were not significantly associated with asthma status in VDAART and were thus not indicative of the overlap in phenotypes. Although this is seemingly counterintuitive given the use of corticosteroid medications to treat asthma [38], this may be due to divergence in the roles of endogenous and exogenous corticosteroids [32]. Our findings showed stronger evidence for a role of metabolites in the androgenic and pregnenolone sub-pathways. The connection between phenotypes in this study suggested that children identified with higher infection proneness through steroid metabolite profiling may be more likely to subsequently develop asthma.

While reduced steroid levels were associated with higher numbers of infections, the mechanism behind this phenomenon is unclear and was not able to be tested with our study design. Future work will incorporate other omics and assess the relationships between steroid metabolites and known inflammatory mediators [39] to further enlighten these associations. There is a known genetic component to infection susceptibility [26], and investigating steroid metabolite profiles with respect to genes that regulate steroidogenesis [40] would provide further insight into the differences in patterns observed across classes, particularly with respect to the restricted analysis results. Replication at the infancy time point in COPSAC may have suffered due to variation in the sample collection procedure; while all other time points were collected in EDTA tubes, COPSAC samples at 18 months were collected in Li-Heparin tubes, and only a few metabolites were replicated in these samples. Despite some limitations, our study represents the first to assess the relationships between steroid metabolites and infection proneness and to attempt to replicate these findings. This is the first step towards the development of a clinically relevant panel to identify infection-prone children. Targeted analysis of steroid metabolites, particularly of the androgenic and pregnenolone classes that showed substantial overlap with the asthma phenotype, should be investigated in follow-up studies to evaluate the utility of this approach as a clinical tool.

## 5. Conclusions

Overall, our study provided evidence of reduced steroid metabolite levels in infection-prone children across multiple time points that were replicated in an independent population. We observed consistent, pronounced relationships between reduced steroids and higher numbers of infections in children, and these associations were robust to differing factors across VDAART and COPSAC cohorts such as race and ethnicity. These results suggested that steroids are important in early life immune susceptibility, and the trends observed during infancy persisted into early school age, indicating that steroid metabolite profiles could be useful tools in early identification of infection-prone children. Overall, the results of this study have important implications for understanding respiratory infection vulnerability and there is a need for further mechanistic study into the role of steroids in infection proneness.

## Figures and Tables

**Table 1:** Demographic and clinical characteristics of child study participants in the VDAART discovery population and COPSAC replication population.

*Discovery Cohort: VDAART*		
Number of subjects	Age 1 Year (n = 449)	Age 6 Years (n = 421)
BMI kg/m^2^, mean (SD)	17.4 (2.2)	16.9 (2.8)
Sex, n (%)		
Female	205 (45.7)	192 (45.6)
Male	244 (54.3)	229 (54.4)
Race, n (%)		
Black	220 (49.0)	207 (49.2)
White	143 (31.8)	133 (31.6)
Other	86 (19.2)	81 (19.2)
Ethnicity, n (%)		
Hispanic or Latino	159 (35.4)	146 (34.7)
Not Hispanic or Latino	290 (64.6)	275 (65.3)
Study site, n (%)		
Boston	139 (31.0)	123 (29.2)
San Diego	149 (33.2)	144 (34.2)
St. Louis	161 (35.9)	154 (36.6)
Oral/Inhaled Corticosteroid Use, n (%)	40 (8.9)	115 (27.3)
Asthma Diagnosis by Age 6, n (%)	121 (26.9)	106 (25.2)
Total Infection Count to Age 6, mean (SD)	28.3 (13.3)	28.3 (12.9)
***Replication cohort: COPSAC***		
Number of subjects	Age 18 Mos. (n = 494)	Age 6 Years (n = 481)
BMI kg/m^2^, mean (SD)	16.4 (2.2)	15.4 (1.3)
Sex, n (%)		
Female	240 (48.6)	224 (46.6)
Male	254 (51.4)	257 (53.4)
Oral/Inhaled Corticosteroid Use, n (%)	76 (15.4)	153 (31.8)
Asthma Diagnosis by Age 6, n (%)	119 (24.1)	105 (21.8)
Total Infection Count to Age 3, mean (SD)	15.2 (9.0)	14.9 (9.2)

**Table 2:** Infection-prone children were more likely to be asthmatic in both VDAART and COPSAC cohorts. Odds ratios, 95% confidence intervals (CIs), and *p*-values are shown for logistic regression models with a dichotomous yes/no for physician diagnosis of asthma by age 6. The lowest quartile of infection proneness was used as the reference group. Models in VDAART were adjusted for sex, race, ethnicity, study site, BMI, and year 6 vitamin D level; models in COPSAC were adjusted for sex, BMI, year 6 vitamin D level, and fish oil intervention status.

Discovery Population: VDAART				
Infection Proneness Quartile	Odds Ratio	95%CI	*p*-Value	% Asthmatics (Total Number)
Quartile 1	-	-	-	13.2 (14)
Quartile 2	1.06	(1.05, 1.06)	0.323	22.6 (24)
Quartile 3	1.05	(1.04, 1.05)	0.448	19.8 (21)
Quartile 4	1.32	(1.31, 1.33)	3.71 × 10^−6^	44.3 (47)
**Replication Population: COPSAC**				
**Infection Proneness Quartile**	**Odds Ratio**	**95%CI**	***p*** **-Value**	**% Asthmatics (Total Number)**
Quartile 1	-	-	-	3.8 (4)
Quartile 2	1.06	(1.06, 1.07)	0.219	16.2 (17)
Quartile 3	1.19	(1.18, 1.19)	1.65 × 10^−3^	23.8 (25)
Quartile 4	1.49	(1.49, 1.50)	1.44 × 10^−13^	56.2 (59)

## Data Availability

The data presented in this study are available upon request. The data are not publicly available due to inclusion of information that could compromise the privacy of research participants.

## Supplementary Materials

The following supporting information can be downloaded at: https://www.mdpi.com/article/10.3390/metabo12111108/s1, Figure S1: Distribution of total number of infections in VDAART and COPSAC cohorts; Table S1: Annotation and biochemical information for 18 steroid metabolites used in analysis, provided by Metabolon; Table S2: Associations between steroid metabolites and respiratory infection proneness in VDAART and COPSAC; Table S3: Restricted analysis of never users of ICS and OCS medications by age 6 years; Table S4: Sex-dependence of associations between steroid metabolites and respiratory infection proneness in VDAART and COPSAC; Table S5: Associations between steroid metabolites at age 6 and asthma status in VDAART. References [24,41] are cited in the supplementary materials
