# Supplementary material for: Reduced Steroid Metabolites Identify Infection-Prone Children in Two Independent Pre-Birth Cohorts
Source: Metabolites. 2022 Nov 13;12(11):1108. doi: 10.3390/metabo12111108 (PMC9692427; doi:10.3390/metabo12111108)
Supplement: Supplementary file 1 [file metabolites-12-01108-s001.zip › metabolites-2006222-supplementary.pdf]

Supplementary Materials

Metabolomic profiling in the Vitamin D Antenatal Asthma Reduction Trial (VDAART) and the Copenhagen Prospective Studies on Asthma in Childhood (COPSAC)

Metabolon ultrahigh-performance liquid chromatography (UPLC) – tandem mass spectrometry (MS/MS) platform method [1]

<u>Sample Preparation:</u> Samples for metabolomic profiling were prepared using the automated MicroLab STAR® system from Hamilton Company. Recovery standards were added prior to the first step in the extraction process for QC purposes. To remove protein, dissociate small molecules bound to protein or trapped in the precipitated protein matrix, and recover chemically diverse metabolites, proteins were precipitated with methanol under vigorous shaking for 2 min (Glen Mills GenoGrinder 2000) followed by centrifugation. The resulting extract was divided into fractions and analyzed by RP/UPLC-MS/MS with negative ion mode ESI to measure steroids. Samples were placed briefly on a TurboVap® (Zymark) to remove organic solvent. The sample extracts were stored overnight under nitrogen prior to analysis.

<u>QA/QC:</u> Controls were analyzed in concert with the experimental samples: a well-characterized, pooled matrix sample generated from a representative, large number of individuals served as a technical replicate throughout the data set; extracted water samples served as process blanks; and a cocktail of QC standards that were carefully chosen not to interfere with the measurement of endogenous compounds were spiked into every analyzed sample to allow instrument performance monitoring and aid in chromatographic alignment. Instrument variability was determined by calculating the median relative standard deviation (RSD) for the standards that were added to each sample prior to injection into the mass spectrometers. Overall process variability was determined by calculating the median RSD for all endogenous metabolites (i.e., non-instrument standards) present in 100% of the pooled matrix samples. Experimental samples were randomized across the platform run with QC samples spaced evenly among the injections.

<u>Ultrahigh Performance Liquid Chromatography – Tandem Mass Spectrometry (UPLC-MS/MS):</u> A Waters ACQUITY ultra-performance liquid chromatography (UPLC) and a Thermo Scientific Q-Exactive high resolution/accurate mass spectrometer interfaced with a heated electrospray ionization (HESI-II) source and Orbitrap mass analyzer operated at 35,000 mass resolution were used for data collection. Samples were reconstituted, and the aliquot was analyzed using basic negative ion optimized conditions using a separate dedicated C18 column. The basic extracts were gradient eluted from the column using methanol and water with 6.5 mM Ammonium Bicarbonate at pH 8. The MS analysis alternated between MS and data-dependent MSn scans using dynamic exclusion. The scan range varied slighted between methods but covered 70-1000 m/z. Raw data files were archived and extracted as described below.

<u>Data Extraction and Compound Identification:</u> Raw data was extracted, peak-identified and QC processed using Metabolon's hardware and software. These systems are built on a web-service platform utilizing Microsoft's .NET technologies, which run on high-performance application servers and fiber-channel storage arrays in clusters to provide active failover and load-balancing. Compounds were identified by comparison to library entries of purified standards or recurrent unknown entities. Metabolon maintains a library based on authenticated standards that contains the retention time/index (RI), mass to charge ratio (m/z), and chromatographic data (including MS/MS spectral data) on all molecules present in the library. Furthermore,

biochemical identifications are based on three criteria: retention index within a narrow RI window of the proposed identification, accurate mass match to the library +/- 10 ppm, and the MS/MS forward and reverse scores between the experimental data and authentic standards. The MS/MS scores are based on a comparison of the ions present in the experimental spectrum to the ions present in the library spectrum. While there may be similarities between these molecules based on one of these factors, the use of all three data points can be utilized to distinguish and differentiate biochemicals. More than 4500 commercially available purified standard compounds have been acquired and registered into LIMS for analysis on all platforms for determination of their analytical characteristics.

Curation: A variety of curation procedures were carried out to ensure that a high quality data set was made available for statistical analysis and data interpretation. The QC and curation processes were designed to ensure accurate and consistent identification of true chemical entities, and to remove those representing system artifacts, mis-assignments, and background noise. Metabolon data analysts use proprietary visualization and interpretation software to confirm the consistency of peak identification among the various samples. Library matches for each compound were checked for each sample and corrected if necessary.

Metabolite Quantification and Data Normalization: Peaks were quantified using area-under-the-curve. A data normalization step was performed to correct variation resulting from instrument inter-day tuning differences by registering the medians to equal one and normalizing each data point proportionately. Datasets were merged by setting the medians of the control groups in the two datasets to be equal. Because sampling was kept consistent across both sample sets, this mitigates concerns that this approach artificially decreases the variance of this control group by forcing the medians to be equal.

Data processing pipeline: We calculated missingness across each metabolite and each sample, according to previously utilized methods [2]. Metabolites missing ≥ 75% were excluded. Missing values were imputed as half the minimum value across all samples for each metabolite. We examined the resulting plots of principal component analysis (PCA). Interquartile range (IQR) and skewness of each metabolite were also computed. All metabolites were subsequently log-10 transformed and pareto-scaled, and IQR and skewness were re-calculated after transformation. PCA was performed again, and distribution of PCs according to demographic variables were examined.

Table S1. *Annotation and biochemical information for 18 steroid metabolites used in analysis, provided by Metabolon.* Information on retention index (RI), biochemical mass, and platform for detection are included to inform annotation for each biochemical by Metabolon. Comp ID and Chemical ID are identifiers used by Metabolon. Additional information is provided, including PubChem Chemical ID, Chemical Abstracts Service (CAS) number, Kyoto Encyclopedia of Genes and Genomes (KEGG) number, Human Metabolome Database (HMDB) number, and Simplified molecular-input line-entry system (SMILES) number.

Comp ID	Biochemical Name	Sub Pathway	Platform	Chemical ID	RI	Mass	SMILES
X38168	16a-hydroxy DHEA 3-sulfate	Androgenic Steroids	LC/MS Neg	100002126	4240	383.1534	O=C1[C@H](O)CC2C3CC=C4C[C@@H](OS(=O)(O)=O)CC[C@]4(C)C3CC[C@@]21C
X37209	androstenediol (3alpha, 17alpha) monosulfate (3)	Androgenic Steroids	LC/MS Neg	100002027	5180	369.1741	O[C@@H]1CCC2C3CCC4=CC(OS(=O)(O)=O)CC[C@]4(C)C3CC[C@@]21C
X37202	androstenediol (3beta,17beta) disulfate (1)	Androgenic Steroids	LC/MS Neg	100001992	3740	224.0618	C[C@@]12C(CCC3C2CC[C@@]4(C)C3CC[C@@H]4OS(=O)(O)=O)=C[C@@H](OS(=O)(O)=O)CC1
X37203	androstenediol (3beta,17beta) disulfate (2)	Androgenic Steroids	LC/MS Neg	100001994	4065	224.0624	
X37211	androstenediol (3beta,17beta) monosulfate (1)	Androgenic Steroids	LC/MS Neg	100002028	4535	369.1741	O[C@H]1CCC2C3CCC4=C[C@@H](OS(=O)(O)=O)CC[C@]4(C)C3CC[C@@]21C
X31591	androsterone sulfate	Androgenic Steroids	LC/MS Neg	100001073	5022	369.1741	C[C@]1([C@](CC2)([H])[C@]3([H])CC[C@@]4([H])C[C@H](OS(=O)(O)=O)CC[C@]4(C)[C@@]3([H])CC1)C2=O
X32425	dehydroepiandrosterone sulfate (DHEA-S)	Androgenic Steroids	LC/MS Neg	100000792	4745	367.1585	[H][C@@]1(CC[C@]2(C)[C@]3(CCC2=O)[H])[C@@]4(C)C(C[C@H](OS(=O)(O)=O)CC4)=CC[C@]13[H]
X33973	epiandrosterone sulfate	Androgenic Steroids	LC/MS Neg	100001287	4855	369.1741	O=C1CCC2C3CC[C@@]4([H])C[C@@H](OS(=O)(O)=O)CC[C@]4(C)C3CC[C@@]21C
X1712	cortisol	Corticosteroids	LC/MS Neg	356	4710	361.202	O[C@@]1([C@@](CO)=O)[C@]2(C)[C@](CC1)([H])[C@@](CCC3=CC4=O)([H])[C@]([C@@]3(C)CC4)([H])[C@@H](O)C2
X1769	cortisone	Corticosteroids	LC/MS Neg	273	4575	359.1864	O[C@@]1([C@@](CO)=O)[C@](C2)(C)[C@](CC1)([H])[C@@](CCC3=CC4=O)([H])[C@]([C@@]3(C)CC4)([H])C2=O
X46115	21-hydroxypregnenolone disulfate	Pregnenolone Steroids	LC/MS Neg	100001999	3935	245.0671	C[C@@]1([C@H]2[C@@](COS(=O)(O)=O)=O)[C@](CC2)([H])[C@@](CC=C3[C@@]4(CC[C@H](OS(=O)(O)=O)C3)C)([H])[C@@]4([H])CC1
X32562	pregnen-diol disulfate*	Pregnenolone Steroids	LC/MS Neg	100001993	3868	238.0775	C[C@@]12C(CCC3C2CC[C@@]4(C)C3CCC4C(OS(=O)(O)=O)C)=CC(OS(=O)(O)=O)CC1
X32619	pregnenediol sulfate (C21H34O5S)*	Pregnenolone Steroids	LC/MS Neg	100002067	5000	397.2054	CC(C1CCC2C3CCC4CC(OS(O)(=O)=O)CCC4(C3CCC12C)C)=O
X38170	pregnenolone sulfate	Pregnenolone Steroids	LC/MS Neg	100002129	5100	395.1898	C[C@]12CC[C@H](OS(=O)(O)=O)CC1=CC[C@@]3([H])[C@]4([H])CC[C@H]([C@](C)=O)[C@](C)4CC[C@]23[H]
X37198	5alpha-pregnan-3beta,20alpha-diol disulfate	Progestin Steroids	LC/MS Neg	100001988	3962	239.0853	C[C@@]12[C@](CCC3C2CC[C@@]4(C)C3CCC4C(OS(=O)(O)=O)C)([H])C[C@@H](OS(=O)(O)=O)CC1
X37200	5alpha-pregnan-3beta,20alpha-diol monosulfate (2)	Progestin Steroids	LC/MS Neg	100002014	5060	399.2211	C[C@@]12[C@](CCC3C2CC[C@@]4(C)C3CCC4[C@](C)([H])O)([H])C[C@@H](OS(=O)(O)=O)CC1
X37196	5alpha-pregnan-3beta,20beta-diol monosulfate (1)	Progestin Steroids	LC/MS Neg	100002009	5180	399.2211	O[C@H]1CC[C@@]2(C)[C@](CCC3C2CC[C@@]4(C)C3CCC4C(C)OS(=O)(O)=O)([H])C1
X46172	5alpha-pregnan-diol disulfate	Progestin Steroids	LC/MS Neg	100002015	4310	239.0853	CC(OS(=O)(O)=O)C1CCC2C3CCC4CC(OS(=O)(O)=O)CCC4(C)C3CCC21C

Supplementary Results

Table S2. *Associations between steroid metabolites and respiratory infection proneness in VDAART and COPSAC.* Incidence rate ratios (IRR), 95% confidence intervals, and P-values are displayed for associations between eighteen steroid metabolites and cumulative respiratory infections. IRRs for metabolites that met the significance threshold of P<0.05 are bolded, and IRRs with P-values between 0.05 and 0.1 are bolded and italicized. Metabolites that were not available in COPSAC at age 18 months or age 6 years are greyed out.

		VDAART YEAR 1 (N=449)				COPSAC 18 MOS (N=494)				VDAART YEAR 6 (N=421)				COPSAC YEAR 6 (N=481)			
**Steroid Metabolite**	**Sub-Pathway**	**IRR**	**95% CI**	**P-Value**	**FDR**	**IRR**	**95% CI**	**P-Value**	**FDR**	**IRR**	**95% CI**	**P-Value**	**FDR**	**IRR**	**95% CI**	**P-Value**	**FDR**
16a-hydroxy DHEA 3-sulfate	Androgenic Steroids	**0.955**	**(0.921, 0.99)**	**0.012**	**0.012**					***0.975***	***(0.949, 1.002)***	***0.065***	***0.078***	**0.965**	**(0.936, 0.994)**	**0.018**	**0.024**
androsterone sulfate	Androgenic Steroids	**0.907**	**(0.873, 0.943)**	**6.42E-07**	**2.89E-06**	0.977	(0.948, 1.006)	0.120	0.211	**0.960**	**(0.933, 0.987)**	**4.59E-03**	**0.012**	**0.933**	**(0.901, 0.966)**	**9.75E-05**	**2.76E-04**
androstenediol (3alpha, 17alpha) monosulfate (3)	Androgenic Steroids	**0.915**	**(0.878, 0.953)**	**2.07E-05**	**4.67E-05**	0.985	(0.954, 1.016)	0.326	0.380	***0.969***	***(0.938, 1.002)***	***0.063***	***0.078***	**0.885**	**(0.849, 0.922)**	**4.28E-09**	**7.28E-08**
androstenediol (3beta,17beta) monosulfate (1)	Androgenic Steroids	**0.939**	**(0.905, 0.974)**	**8.27E-04**	**9.31E-04**					***0.978***	***(0.953, 1.004)***	***0.097***	0.110	**0.940**	**(0.917, 0.963)**	**8.11E-07**	**6.90E-06**
androstenediol (3beta,17beta) disulfate (1)	Androgenic Steroids	**0.930**	**(0.897, 0.963)**	**5.29E-05**	**1.06E-04**	**0.969**	**(0.943, 0.995)**	**0.022**	***0.061***	**0.969**	**(0.941, 0.998)**	**0.035**	***0.053***	**0.933**	**(0.903, 0.964)**	**3.37E-05**	**1.43E-04**
androstenediol (3beta,17beta) disulfate (2)	Androgenic Steroids	**0.925**	**(0.889, 0.963)**	**1.65E-04**	**2.29E-04**	**0.948**	**(0.914, 0.985)**	**5.46E-03**	**0.025**	**0.939**	**(0.908, 0.97)**	**1.78E-04**	**1.60E-03**	**0.925**	**(0.891, 0.96)**	**4.94E-05**	**1.68E-04**
dehydroepiandrosterone sulfate (DHEA-S)	Androgenic Steroids	**0.922**	**(0.889, 0.957)**	**1.47E-05**	**4.40E-05**	**0.954**	**(0.924, 0.984)**	**3.33E-03**	**0.023**	**0.966**	**(0.936, 0.996)**	**0.029**	**0.047**	**0.917**	**(0.884, 0.951)**	**3.36E-06**	**1.90E-05**
epiandrosterone sulfate	Androgenic Steroids	**0.941**	**(0.908, 0.974)**	**6.63E-04**	**7.96E-04**	***0.973***	***(0.945, 1.001)***	***0.062***	0.144	***0.973***	***(0.945, 1.001)***	***0.059***	***0.078***	**0.936**	**(0.904, 0.97)**	**2.39E-04**	**5.08E-04**
cortisone	Corticosteroids	**0.811**	**(0.765, 0.859)**	**1.17E-12**	**2.09E-11**	0.971	(0.917, 1.027)	0.305	0.380	**0.947**	**(0.908, 0.986)**	**9.13E-03**	**0.018**	***0.946***	***(0.896, 1)***	***0.050***	***0.057***
cortisol	Corticosteroids	**0.824**	**(0.780, 0.870)**	**2.33E-12**	**2.09E-11**	**1.060**	**(1.01, 1.114)**	**0.019**	***0.061***	**0.945**	**(0.908, 0.984)**	**5.73E-03**	**0.013**	1.000	(0.946, 1.057)	0.991	0.991
pregnenolone sulfate	Pregnenolone Steroids	**0.899**	**(0.861, 0.939)**	**1.48E-06**	**5.31E-06**					**0.949**	**(0.921, 0.978)**	**6.20E-04**	**2.79E-03**	**0.935**	**(0.904, 0.968)**	**1.28E-04**	**3.12E-04**
pregnen-diol disulfate#	Pregnenolone Steroids	**0.911**	**(0.872, 0.951)**	**2.02E-05**	**4.67E-05**	0.972	(0.929, 1.017)	0.215	0.302	**0.947**	**(0.913, 0.982)**	**3.09E-03**	**9.27E-03**	**0.949**	**(0.907, 0.992)**	**0.022**	**0.026**
pregnenediol sulfate (C21H34O5S)#	Pregnenolone Steroids	**0.867**	**(0.828, 0.908)**	**1.09E-09**	**6.57E-09**	0.967	(0.923, 1.013)	0.154	0.240	**0.954**	**(0.919, 0.99)**	**0.012**	**0.022**	**0.921**	**(0.88, 0.964)**	**3.72E-04**	**6.32E-04**
21-hydroxypregnenolone disulfate	Pregnenolone Steroids	**0.930**	**(0.893, 0.969)**	**5.62E-04**	**7.23E-04**	**0.940**	**(0.905, 0.977)**	**1.55E-03**	**0.022**	**0.926**	**(0.892, 0.96)**	**4.05E-05**	**7.29E-04**	0.971	(0.935, 1.008)	0.125	0.133
5alpha-pregnan-diol disulfate	Progestin Steroids	**0.937**	**(0.906, 0.969)**	**1.55E-04**	**2.29E-04**					0.982	(0.955, 1.009)	0.181	0.181				
5alpha-pregnan-3beta,20beta-diol monosulfate (1)	Progestin Steroids	**0.919**	**(0.881, 0.959)**	**1.11E-04**	**2.00E-04**	1.005	(0.972, 1.04)	0.759	0.759	0.977	(0.949, 1.005)	0.111	0.117	**0.940**	**(0.908, 0.972)**	**3.42E-04**	**6.32E-04**
5alpha-pregnan-3beta,20alpha-diol monosulfate (2)	Progestin Steroids	**0.944**	**(0.908, 0.981)**	**3.20E-03**	**3.38E-03**	0.976	(0.947, 1.005)	0.105	0.210	**0.951**	**(0.922, 0.981)**	**1.45E-03**	**5.21E-03**	**0.951**	**(0.915, 0.988)**	**9.68E-03**	**0.014**
5alpha-pregnan-3beta,20alpha-diol disulfate	Progestin Steroids	**0.920**	**(0.881, 0.961)**	**1.65E-04**	**2.29E-04**	0.988	(0.946, 1.032)	0.592	0.637	**0.936**	**(0.903, 0.971)**	**4.10E-04**	**2.46E-03**	**0.936**	**(0.899, 0.975)**	**1.31E-03**	**2.03E-03**

Incidence rate ratios and P-values are shown based on Poisson regression models adjusted for sex, race#, ethnicity#, study site#, BMI, steroid medication use within 3 months of blood draw, and year 6 vitamin D level (# = covariate only in VDAART). Fish oil intervention status was also included as a covariate for COPSAC only.

Table S3. *Sex-dependence of associations between steroid metabolites and respiratory infection proneness in VDAART and COPSAC.* Interactions between sex and steroid metabolites were assessed in regression models of associations between steroid metabolites and respiratory infection proneness, with females as the referent group. Interaction coefficients and P-values are displayed for the eighteen steroid metabolites. Models were adjusted for race#, ethnicity#, study site#, BMI, steroid medication use within 3 months of blood draw, and year 6 vitamin D level (# = covariate only in VDAART). Interaction coefficients that met the significance threshold of P<0.05 are bolded; interaction coefficients with P-values between 0.05 and 0.1 are bolded and italicized. Metabolites that were not available in COPSAC at age 6 years are greyed out.

	**VDAART Age 6 (N=421)**			**COPSAC Age 6 (N=481)**		
Steroid Metabolite	**Estimate**	**95% CI**	**P-Value**	**Estimate**	**95%CI**	**P-Value**
16a-hydroxy DHEA 3-sulfate	**0.947**	**(0.944, 0.949)**	**0.043**	0.953	(0.950, 0.955)	0.115
androsterone sulfate	**0.921**	**(0.918, 0.923)**	**4.19E-03**	***0.934***	***(0.931, 0.938)***	***0.062***
androstenediol (3beta,17alpha) monosulfate (3)	**0.905**	**(0.902, 0.908)**	**2.81E-03**	***0.926***	***(0.923, 0.930)***	***0.076***
androstenediol (3beta,17beta) monosulfate (1)	**0.903**	**(0.901, 0.905)**	**6.66E-05**	0.967	(0.965, 0.969)	0.185
androstenediol (3beta,17beta) disulfate (1)	**0.921**	**(0.919, 0.924)**	**4.02E-03**	**0.912**	**(0.909, 0.914)**	**5.57E-03**
androstenediol (3beta,17beta) disulfate (2)	0.958	(0.955, 0.961)	0.199	**0.926**	**(0.923, 0.929)**	**0.046**
dehydroepiandrosterone sulfate (DHEA-S)	**0.894**	**(0.891, 0.896)**	**2.75E-04**	**0.906**	**(0.903, 0.909)**	**8.33E-03**
epiandrosterone sulfate	**0.924**	**(0.922, 0.927)**	**6.29E-03**	**0.900**	**(0.897, 0.902)**	**5.14E-03**
cortisone	**0.896**	**(0.892, 0.900)**	**4.64E-03**	**0.880**	**(0.875, 0.884)**	**0.027**
cortisol	**0.926**	**(0.922, 0.929)**	**0.039**	**0.754**	**(0.750, 0.758)**	**1.83E-06**
pregnenolone sulfate	***0.945***	***(0.942, 0.948)***	***0.055***	0.963	(0.960, 0.966)	0.290
pregnen-diol disulfate#	***0.940***	***(0.936, 0.943)***	***0.092***	**0.911**	**(0.908, 0.915)**	**0.045**
pregnenediol sulfate ($C_{21}H_{34}O_5S$)#	0.960	(0.957, 0.963)	0.272	**0.892**	**(0.889, 0.896)**	**0.015**
21-hydroxypregnenolone disulfate	***0.932***	***(0.929, 0.935)***	***0.064***	**0.898**	**(0.895, 0.902)**	**5.68E-03**
5alpha-pregnan-diol disulfate	**0.914**	**(0.912, 0.916)**	**1.09E-03**			
5alpha-pregnan-3beta,20beta-diol monosulfate (1)	***0.948***	***(0.946, 0.951)***	***0.070***	1.011	(1.007, 1.014)	0.762
5alpha-pregnan-3beta,20alpha-diol monosulfate (2)	1.003	(1.000, 1.006)	0.928	**0.856**	**(0.853, 0.859)**	**1.69E-04**
5alpha-pregnan-3beta,20alpha-diol disulfate	0.947	(0.943, 0.950)	0.141	0.950	(0.947, 0.954)	0.234

Models were adjusted for sex, race#, ethnicity#, study site#, BMI, steroid medication use within 3 months of blood draw, and year 6 vitamin D level (# = covariate only in VDAART). COPSAC models included additional adjustment for fishoil intervention.

Distribution of respiratory infection proneness variable in VDAART and COPSAC populations

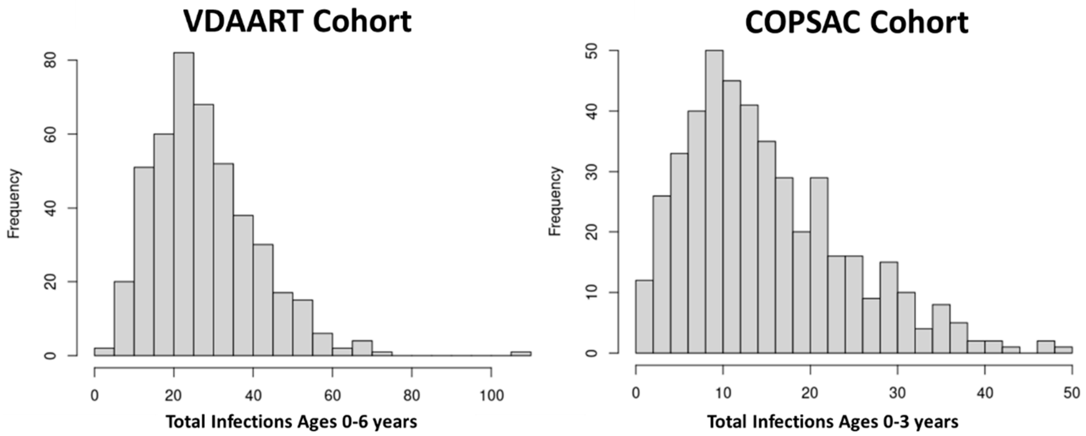

Figure S1. *Distribution of total number of infections in VDAART and COPSAC cohorts.* The cumulative number of respiratory infections that occurred between birth and age 6 years (VDAART) or birth and age 3 years (COPSAC) was used as a proxy to estimate respiratory infection proneness of the children.

Table S4. *Restricted analysis of never users of ICS and OCS medications by age 6 years.* Incidence rate ratios (IRR), 95% confidence intervals, and P-values are displayed for associations between eighteen steroid metabolites and cumulative respiratory infections in non-users of ICS/OCS. IRRs for metabolites that met the significance threshold of P<0.05 are bolded, and IRRs with P-values between 0.05 and 0.1 are bolded and italicized. Metabolites that were not available in COPSAC at age 18 months or age 6 years are greyed out. Models were adjusted for same covariates as original models, except steroid medication use variable.

		VDAART YEAR 6 (N=306)				COPSAC YEAR 6 (N=328)			
**Steroid Metabolite**	**Sub-Pathway**	**IRR**	**95% CI**	**P-Value**	**FDR**	**IRR**	**95% CI**	**P-Value**	**FDR**
16a-hydroxy DHEA 3-sulfate	Androgenic Steroids	***0.968***	***(0.934, 1.003)***	***0.070***	***0.079***	**0.944**	**(0.908, 0.982)**	**4.08E-03**	**0.012**
androsterone sulfate	Androgenic Steroids	**0.919**	**(0.888, 0.952)**	**2.93E-06**	**2.14E-05**	**0.947**	**(0.904, 0.992)**	**0.021**	**0.036**
androstenediol (3alpha, 17alpha) monosulfate (3)	Androgenic Steroids	**0.912**	**(0.877, 0.948)**	**3.57E-06**	**2.14E-05**	**0.928**	**(0.880, 0.979)**	**6.49E-03**	**0.016**
androstenediol (3beta,17beta) monosulfate (1)	Androgenic Steroids	**0.967**	**(0.936, 0.999)**	**0.043**	**0.053**	**0.961**	**(0.931, 0.993)**	**0.018**	**0.035**
androstenediol (3beta,17beta) disulfate (1)	Androgenic Steroids	**0.936**	**(0.903, 0.970)**	**2.71E-04**	**6.98E-04**	**0.921**	**(0.883, 0.960)**	**1.18E-04**	**8.04E-04**
androstenediol (3beta,17beta) disulfate (2)	Androgenic Steroids	**0.921**	**(0.885, 0.960)**	**7.74E-05**	**2.32E-04**	**0.921**	**(0.877, 0.968)**	**1.28E-03**	**5.45E-03**
dehydroepiandrosterone sulfate (DHEA-S)	Androgenic Steroids	**0.954**	**(0.918, 0.992)**	**0.017**	**0.026**	**0.908**	**(0.865, 0.953)**	**9.49E-05**	**8.04E-04**
epiandrosterone sulfate	Androgenic Steroids	**0.931**	**(0.899, 0.964)**	**5.77E-05**	**2.08E-04**	0.966	(0.923, 1.012)	0.147	0.166
cortisone	Corticosteroids	0.976	(0.925, 1.030)	0.383	0.383	**1.105**	**(1.017, 1.201)**	**0.019**	**0.035**
cortisol	Corticosteroids	**0.936**	**(0.887, 0.987)**	**0.015**	**0.024**	**1.170**	**(1.079, 1.269)**	**1.42E-04**	**8.04E-04**
pregnenolone sulfate	Pregnenolone Steroids	0.976	(0.940, 1.012)	0.186	0.197	***0.957***	***(0.916, 1.00)***	***0.052***	***0.080***
pregnen-diol disulfate[#]	Pregnenolone Steroids	**0.907**	**(0.869, 0.946)**	**7.19E-06**	**3.24E-05**	0.956	(0.920, 1.013)	0.126	0.154
pregnenediol sulfate (C21H34O5S)[#]	Pregnenolone Steroids	**0.954**	**(0.911, 0.999)**	**0.044**	**0.053**	**0.916**	**(0.862, 0.973)**	**4.39E-03**	**0.012**
21-hydroxypregnenolone disulfate	Pregnenolone Steroids	**0.919**	**(0.878, 0.963)**	**3.69E-04**	**7.37E-04**	0.960	(0.913, 1.010)	0.113	0.148
5alpha-pregnan-diol disulfate	Progestin Steroids	**0.943**	**(0.913, 0.974)**	**3.69E-04**	**7.37E-04**				
5alpha-pregnan-3beta,20beta-diol monosulfate (1)	Progestin Steroids	**0.961**	**(0.927, 0.996)**	**0.030**	**0.042**	0.971	(0.929, 1.015)	0.198	0.210
5alpha-pregnan-3beta,20alpha-diol monosulfate (2)	Progestin Steroids	**0.937**	**(0.902, 0.975)**	**1.16E-03**	**2.08E-03**	0.983	(0.933, 1.036)	0.517	0.517
5alpha-pregnan-3beta,20alpha-diol disulfate	Progestin Steroids	**0.880**	**(0.842, 0.919)**	**1.05E-08**	**1.89E-07**	0.958	(0.911, 1.008)	0.101	0.143

Table S5. *Associations between steroid metabolites and age 6 and asthma status in VDAART.* Odds ratios (ORs), 95% confidence intervals, and P-values are displayed for associations between eighteen steroid metabolites and asthma diagnosis by age 6 years. ORs for metabolites that met the significance threshold of P<0.1 are bolded. Metabolites that also met nominal significance at P<0.05 for infection proneness are shown in grey.

		VDAART YEAR 6 (N=421)		
**Steroid Metabolite**	**Sub-Pathway**	**OR**	**95% CI**	**P-Value**
16a-hydroxy DHEA 3-sulfate	Androgenic Steroids	**0.69**	**(0.45, 1.07)**	**0.100**
androsterone sulfate	Androgenic Steroids	**0.46**	**(0.29, 0.71)**	**5.68E-04**
androstenediol (3alpha, 17alpha) monosulfate (3)	Androgenic Steroids	**0.44**	**(0.26, 0.73)**	**1.46E-03**
androstenediol (3beta,17beta) monosulfate (1)	Androgenic Steroids	**0.54**	**(0.36, 0.80)**	**2.36E-03**
androstenediol (3beta,17beta) disulfate (1)	Androgenic Steroids	**0.54**	**(0.34, 0.85)**	**8.73E-03**
androstenediol (3beta,17beta) disulfate (2)	Androgenic Steroids	**0.62**	**(0.37, 1.04)**	**0.068**
dehydroepiandrosterone sulfate (DHEA-S)	Androgenic Steroids	**0.46**	**(0.28, 0.76)**	**2.33E-03**
epiandrosterone sulfate	Androgenic Steroids	**0.50**	**(0.32, 0.78)**	**2.44E-03**
cortisone	Corticosteroids	1.26	(0.64, 2.50)	0.49
cortisol	Corticosteroids	1.00	(0.54, 1.8)	0.99
pregnenolone sulfate	Pregnenolone Steroids	0.72	(0.45, 1.15)	0.172
pregnen-diol disulfate#	Pregnenolone Steroids	**0.50**	**(0.28, 0.90)**	**0.0201**
pregnenediol sulfate (C21H34O5S)#	Pregnenolone Steroids	**0.44**	**(0.24, 0.82)**	**9.11E-03**
21-hydroxypregnenolone disulfate	Pregnenolone Steroids	**0.60**	**(0.34, 1.08)**	**0.0878**
5alpha-pregnan-diol disulfate	Progestin Steroids	0.71	(0.44, 1.13)	0.150
5alpha-pregnan-3beta,20beta-diol monosulfate (1)	Progestin Steroids	0.70	(0.45, 1.10)	0.125
5alpha-pregnan-3beta,20alpha-diol monosulfate (2)	Progestin Steroids	0.68	(0.42, 1.09)	0.109
5alpha-pregnan-3beta,20alpha-diol disulfate	Progestin Steroids	0.71	(0.40, 1.28)	0.258

Models were adjusted for sex, race, ethnicity, study site, BMI, and year 6 vitamin D level.

References

1. Evans AM, DeHaven CD, Barrett T, Mitchell M, Milgram E. Integrated, nontargeted ultrahigh performance liquid chromatography/electrospray ionization tandem mass spectrometry platform for the identification and relative quantification of the small-molecule complement of biological systems. *Anal Chem*. Aug 15 2009;81(16):6656-67. doi:10.1021/ac901536h
2. Huang M, Kelly RS, Chu SH, et al. Maternal Metabolome in Pregnancy and Childhood Asthma or Recurrent Wheeze in the Vitamin D Antenatal Asthma Reduction Trial. *Metabolites*. Jan 23 2021;11(2)doi:10.3390/metabo11020065
